# Opiate Injection–Associated Skin, Soft Tissue, and Vascular Infections, England, UK, 1997–2016

**DOI:** 10.3201/eid2308.170439

**Published:** 2017-08

**Authors:** Dan Lewer, Magdalena Harris, Vivian Hope

**Affiliations:** Public Health England, London, UK (D. Lewer);; London School of Hygiene and Tropical Medicine, London (M. Harris);; Liverpool John Moores University, Liverpool, UK (V. Hope)

**Keywords:** Substance abuse intravenous, soft tissue infections, bacterial infections, vascular infections, skin infections, injection drug use, bacteria, opiates, England, United Kingdom

## Abstract

In England, UK, hospital admissions caused by bacterial infections associated with opioid use have increased annually since 2012, after 9 years of decline, mirroring trends in overdose deaths. The increase occurred among persons of both sexes and in all age groups and suggests preventive measures need reviewing.

In the United Kingdom, opioid overdose deaths have increased substantially, linked to increasing purity of street heroin and an aging cohort of persons who inject drugs (PWID) (*1*). PWID also are at risk for skin, soft tissue, and vascular infections (SSTVI), and one third of PWID in the United Kingdom report symptoms of an injection-site infection within the previous year (*2*). Outbreaks and clusters of bacterial infections among PWID are documented in the United Kingdon (*3**,**4*). Most infections are caused by staphylococci and other commensal gram-positive bacteria entering the body at injecting sites. Abscesses and phlebitis are common (*5**,**6*) and can lead to invasive infections. Data from a London hospital suggest that such skin and soft tissue infections cause 58% of hospital admissions in PWID, and treatment typically costs several times more than infections in other groups (*5*). Because little is known about SSTVI trends among PWID over time, we used routine data from all National Health Service hospitals in England to describe hospital admissions for this group.

## The Study

We used the Hospital Episode Statistics for England dataset and included all admissions from April 5, 1997, through April 4, 2016, for patients 15–55 years of age. As the most common injecting-related problems (*6*), we included admissions with a primary (or first-listed) cause of cutaneous abscess (International Classification of Diseases, Tenth Revision, code L02*), cellulitis (L03*), and phlebitis or thrombophlebitis (I80*). We also included admissions where the first-listed cause was endocarditis (I011, I39*, I330, 1400, I410), septicemia (A40*, A41*), osteomyelitis or septic arthritis (M86*, M00*, M465), or necrotizing fasciitis (M762) and grouped these as invasive infections. Because patients might have multiple episodes of care within 1 admission, we included only first episodes. Age, sex, year of admission, all diagnostic fields, and duration of admission were extracted. Public Health England provided the data.

Hospital Episode Statistics do not report whether a patient injects drugs. Previous studies have identified patients who use drugs as those with a drug-related diagnosis in any diagnostic field (*7**,**8*). We identified patients with “injecting-related” infections as those with a relevant infection in the primary diagnostic field and “mental and behavioral disorders due to opioid use” (F11*) in any other diagnostic field, because most PWID in the United Kingdom inject an opioid (*9*). 

We counted injecting-related and non–injecting-related admissions and stratified them by year and patient sex and age group (15–34, 35–44, and 45–55 years). We also tested whether injecting-related infections were associated with longer hospitalization by using a zero-inflated negative binomial model (*10*) (Technical Appendix).

During 1997–2016, a total of 1,052,444 hospital admissions were caused by SSTVIs, of which 63,671 (6%) were injecting-related. One third (35%) of injecting-related admissions had a primary cause of cutaneous abscess, 32% phlebitis, 23% cellulitis, 4% septicemia, 4% osteomyelitis or septic arthritis, 2% endocarditis, and 0.2% necrotizing fasciitis. Patients with injecting-related infections were younger and more likely to live in deprived neighborhoods, and a minority were female (Table).

**Table:** Demographic characteristics of patients with and without injecting-related infections, England, UK, April 5, 1997–April 4, 2016*

Characteristic	Patients with injecting-related infections	Patients with non–injecting-related infections
Median age, y (IQR)		
All	34 (29–39)	40 (30–48)
M	34 (30–40)	40 (31–48)
F	32 (27–37)	39 (29–48)
By year		
2000–01	31 (27–36)	39 (30–48)
2005–06	32 (28–37)	39 (30–47)
2010–11	35 (30–41)	40 (30–48)
2015–16	38 (33–43)	41 (30–49)
Female sex, %		
All	28	44
By age group, y		
15–34	32	45
35–44	23	42
45–54	22	43
Neighborhood deprivation quintile, %		
1 (least deprived)	5	21
2	10	21
3	16	20
4	25	20
5 (most deprived)	44	19

*Selected years are shown for brevity. Patients with injecting-related infections were younger for both sexes and in each year (p<0.001, Wilcoxon rank-sum tests). A smaller proportion of patients with injecting-related infections were female for all age groups (p<0.001, χ^2^ tests). Age group was associated with sex for both injecting-related and non–injecting-related infections (p<0.001, χ^2^ tests). A linear trend described the proportion of injecting-related admissions in each deprivation quintile better than no trend (p = 0.009) but not for non–injecting-related admission (p = 0.504). Neighborhood deprivation was the UK Department for Community and Local Government’s Index of Multiple Deprivation 2004. IQR, interquartile range.

**Figure 1:**
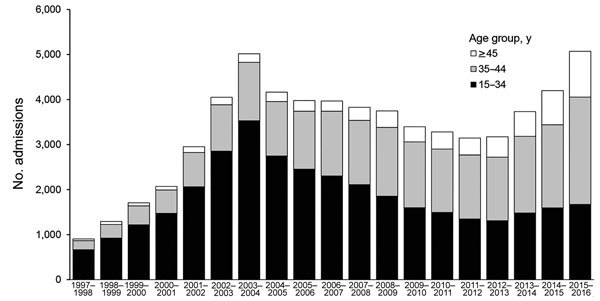
Number of hospital admissions caused by injecting-related bacterial infections, by age group, England, UK, April 5, 1997–April 4, 2016.

The number of injecting-related admissions increased by 33% per year (compound annual growth rate) from 1997–98 through 2003–04 (Figure 1); relative increases were similar in each age group. The total number then decreased each year from 2003–04 through 2012–13; relative changes differed by age group. Admissions reduced by 15% per year for 15–34-year-olds, remained approximately constant for 35–44-year-olds, and increased by 5% per year for 45–55-year-olds. From 2012–13 through 2015–16, the total number of injecting-related admissions increased each year in all age groups. The largest relative increase was for 45–55-year-olds (18% per year). The number of non–injecting-related admissions increased throughout the period; relative increases were similar for each age group and for men and women (Technical Appendix Figure 1).

As a sensitivity analysis, we excluded admissions within 7 days after discharge, which totaled 4,389 (7%) injecting-related admissions. This exclusion did not change the overall trend (Technical Appendix).

**Figure 2:**
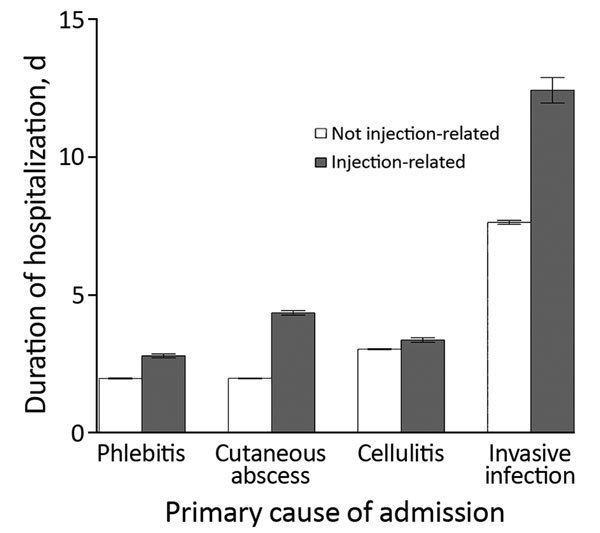
Modeled duration of hospitalization for men 35–44 years of age, by cause of admission, England, UK, April 5, 1997–April 4, 2016. Hospitalization duration was longer for injecting-related admissions for all causes (p<0.001). Error bars indicate 95% CIs.

Injecting-related admissions were longer than non–injecting-related admissions. The difference varied by cause of admission; differences were larger for admissions caused by cutaneous abscess or by invasive infections (Figure 2).

## Conclusions

Our analysis of hospital data shows a substantial increase in episodes of serious infection among PWID since 2012. Increases occurred in all age groups and for both men and women. Community surveys have not indicated such a large increase in the prevalence of symptoms of injection-site infections (*9*), suggesting that the increase might be confined to more severe infections.

The temporal trend found here for bacterial infections mirrors that for opiate overdose–related deaths in England and Wales, which increased sharply from the early 1990s until 2001, decreased gradually until 2012, and then increased again (*1*). Explanations given for the recent increase in overdoses include an aging cohort of PWID, increasing purity of street heroin, and an increased focus by addiction services on treatment completions, including reducing the number of patients on long-term opioid substitution therapy (*1**,**11*). These factors also might contribute to the increase in bacterial infections. Older PWID may lose venous access; miss veins more often when trying to inject (*12*); and use less accessible and more heavily colonized injection sites, such as the femoral vein (*13*), leading to more infections. These persons also might have worse immunity and poorer underlying health. An aging cohort of PWID is unlikely to explain the entire increase, however, because increases occurred in all age groups. The role of changes to addiction services and street heroin purity are potential areas for further research.

Additional factors might be contributing to the increase. Opiate users may have started to inject recently emerged psychoactive drugs, which are associated with increased risk for serious bacterial infection (*14*), although the injection of these drugs remains relatively uncommon in the United Kingdom (*9*). Primary care services might have become less accessible to PWID, leading to a greater proportion of infections becoming serious and requiring hospitalization. In London, drug preparation using citric acid has been documented to result in highly acidic heroin mixtures (*15*), potentially precipitating venous damage and infections. Finally, the increase in infections could indicate that the population of PWID has grown since 2012, but little evidence exists with which to test this possibility.

A limitation of our study is that Hospital Episode Statistics do not record whether patients inject drugs, and therefore a proxy was used. The data are likely to underestimate the true number of SSTVI in PWID because hospitals might not always include the opioid-related diagnostic code when PWID are admitted.

Illnesses and deaths from bacterial infections in PWID are more difficult to measure than overdoses because bacterial infections are not specific to drug use. The increasing number of serious infections shown by these data suggests a need for more active surveillance. Preventive measures also need to be considered, including improving access and adherence to wound care and antimicrobial drug regimens, reducing the acidity of heroin preparations, and ensuring accessibility of opioid substitution therapy and sterile injecting equipment.

Technical Appendix Duration and number of hospital admissions with a primary cause relating to bacterial infection; and sensitivity analysis, England, UK, April 5, 1997–April 4, 2016.
