# Supplementary material for: Opiate Injection–Associated Skin, Soft Tissue, and Vascular Infections, England, UK, 1997–2016
Source: Emerg Infect Dis. 2017 Aug;23(8):1400–3. doi: 10.3201/eid2308.170439 (PMC5547794; doi:10.3201/eid2308.170439)
Supplement: Technical Appendix — Duration and number of hospital admissions with a primary cause relating to bacterial infection; and sensitivity analysis, England, UK, April 5, 1997–April 4, 2016. [file 17-0439-Techapp-s1.pdf]

Article DOI: https://doi.org/10.3201/eid2308.170439

Opiate Injection–Associated Skin, Soft Tissue, and Vascular Infections, England, UK, 1997–2016

Technical Appendix

Duration of Hospitalization

The duration of hospitalization is measured in days. Twenty-one percent of admissions were 0 days (i.e., discharge was on the same day as admission). The data are right-skewed with some extreme values (up to 761 days). We fitted a zero-inflated negative binomial model to explore predictors of admission duration. This approach was selected because there was strong evidence of overdispersion in a Poisson model. The dependent variable was the duration of admission (as a continuous variable in days), and the independent variables were the primary cause of admission (cutaneous abscess, phlebitis, cellulitis, or invasive infections, defined as a primary [first-listed] cause of septicemia, osteomyelitis or septic arthritis, endocarditis, and necrotizing fasciitis), whether the infection was injecting–related (a binary indicator), the patient’s age (15–34, 35–44, and 45–55 years), and an interaction term between the cause of admission and injecting status. The primary cause of admission was used to predict whether admissions were 0 days in the logit part of the model. The exponentiated coefficients can be interpreted as the ratio of days stayed in hospital when comparing groups.

The results are shown in the Technical Appendix Table. The table shows the stratum-specific effects of injecting status rather than the interactions. Compared with cutaneous abscess, admissions due to phlebitis, cellulitis, or invasive infections were associated with longer stays. Older age and male sex were associated with longer stays. Injecting-related infections were associated with longer stays, and this effect varied according to the cause of admission. We used the model to predict the length of admission for men aged 35–44 years (as the largest group of injecting-related admissions in 2015–16), stratifying by primary cause of admission and injecting

status, with bootstrapped confidence intervals. These predicted values are shown in Figure 2 in the main article.

Sensitivity Analysis Excluding Closely Spaced Admissions

We conducted a sensitivity analysis excluding "repeat admissions," defined as having an admission date ≤7 days after discharge from a previous admission. These admissions may be for the same infection rather than a new incident infection. A total of 4,389 injecting-related admissions were excluded (7% of all injecting-related admissions). This did not change the overall trend (Technical Appendix Figure 2).

Technical Appendix Table. Incident rate ratio of days stayed in hospital (fully adjusted), England, UK, April 5, 1997–April 4, 2016

Variable and level	Exponentiated regression coefficient (95% CI)	p value
Primary cause of admission*		
Cutaneous abscess (ref)	1	
Phlebitis	1.95 (1.93–1.98)	<0.001
Cellulitis	1.54 (1.53–1.55)	<0.001
Invasive infection	4.05 (4.01–4.08)	<0.001
Age, y		
15–34 (ref)	1	
35–44	1.18 (1.17–1.19)	<0.001
45–55	1.35 (1.34–1.36)	<0.001
Sex		
M (ref)	1	
F	0.91 (0.91–0.92)	<0.001
Injecting-related (ref = "no")		
Cutaneous abscess	2.22 (2.17–2.26)	<0.001
Phlebitis	1.42 (1.38–1.46)	<0.001
Cellulitis	1.11 (1.08–1.14)	<0.001
Invasive infection†	1.63 (1.57–1.69)	<0.001

*Causes are cutaneous abscess, phlebitis, cellulitis, septicemia, osteomyelitis or septic arthritis, endocarditis, and necrotizing fasciitis. See main article for details.
†Invasive infections are defined as those with a primary (first-listed) cause of septicemia, osteomyelitis or septic arthritis, endocarditis, and necrotizing fasciitis.

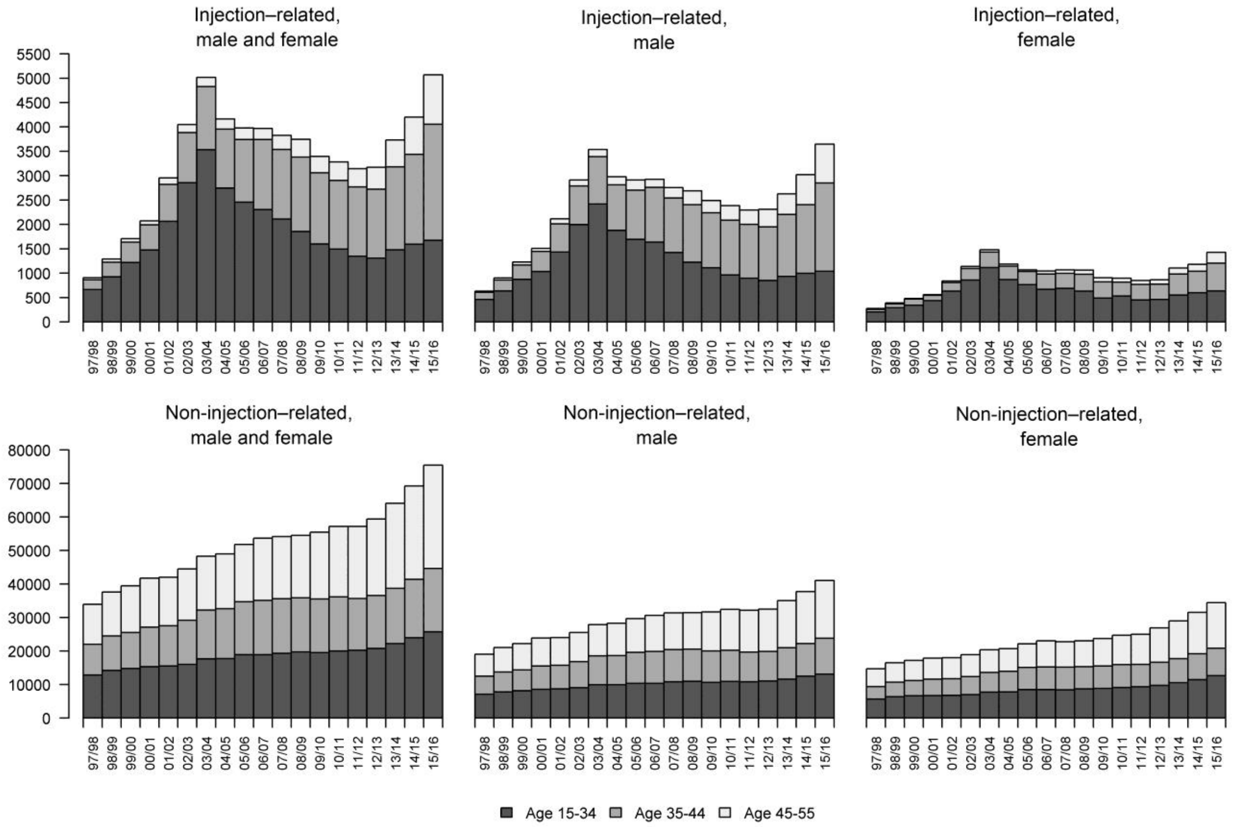

Technical Appendix Figure 1. Number of hospital admissions with a primary cause relating to bacterial infection, by sex, age group, and injecting status, England, UK, April 5, 1997–April 4, 2016. Invasive infections are defined as those with a primary (first-listed) cause of septicemia, osteomyelitis or septic arthritis, endocarditis, and necrotizing fasciitis. Causes are cutaneous abscess, phlebitis, cellulitis, septicemia, osteomyelitis or septic arthritis, endocarditis, and necrotizing fasciitis. See main article for details.

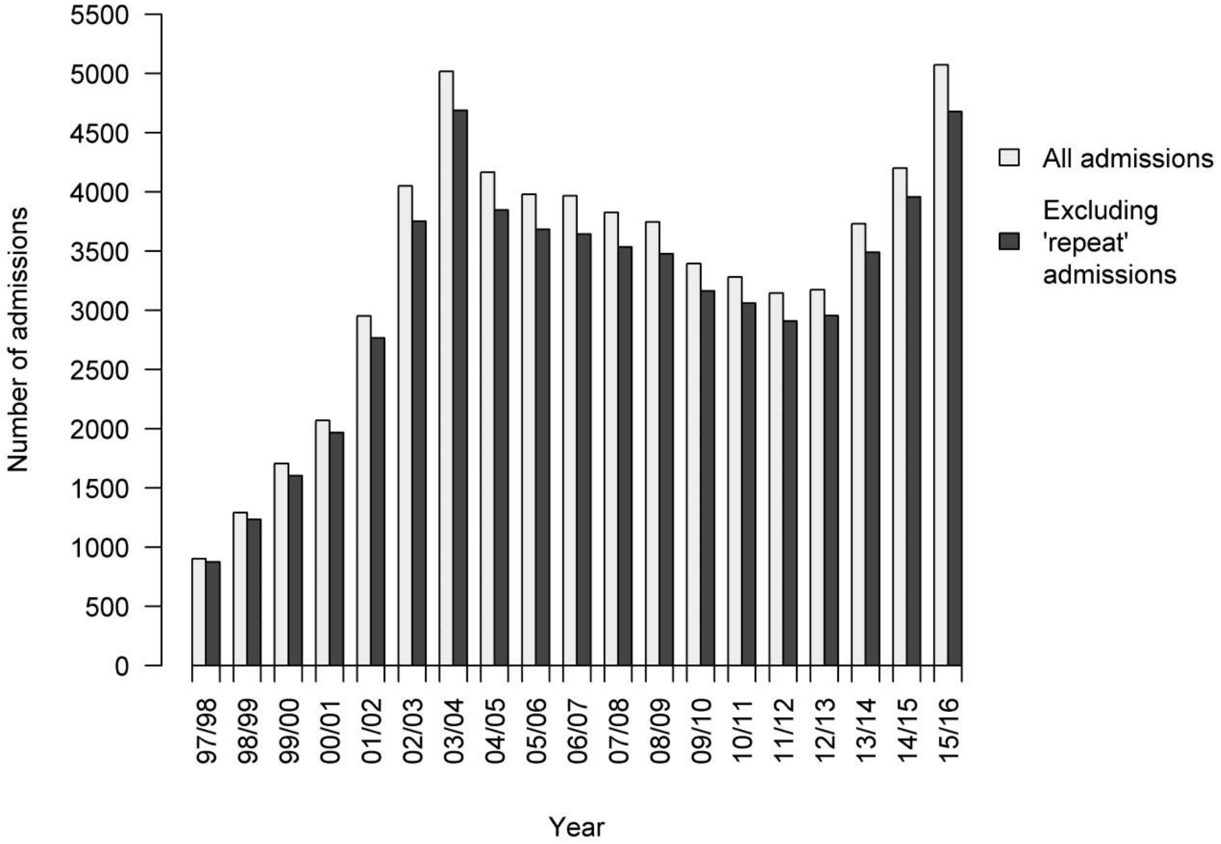

Technical Appendix Figure 2. Number of hospital admissions due to injecting-related bacterial infections, England, UK, April 5, 1997–April 4, 2016. “Repeat” admissions are defined as those occurring ≤7 days after discharge from a previous admission.
